# Diel Variations in Cell Abundance and Trophic Transfer of Diarrheic Toxins during a Massive *Dinophysis* Bloom in Southern Brazil

**DOI:** 10.3390/toxins10060232

**Published:** 2018-06-06

**Authors:** Thiago Pereira Alves, Luiz Laureno Mafra

**Affiliations:** 1 Center for Marine Studies, Federal University of Paraná. Av. Beira-mar s/n, P.O. Box: 61, Pontal do Paraná PR 83255-976, Brazil; 2 Federal Institute of Santa Catarina, Av. Ver. Abraão João Francisco, 3988, Ressacada, Itajaí SC 88307-303, Brazil

**Keywords:** harmful algal bloom, Diarrheic Shellfish Poisoning, okadaic acid, toxin accumulation, toxin vectors, trophic transfer, Brazil

## Abstract

*Dinophysis* spp. are a major source of diarrheic toxins to marine food webs, especially during blooms. This study documented the occurrence, in late May 2016, of a massive toxic bloom of the *Dinophysis acuminata* complex along the southern coast of Brazil, associated with an episode of marked salinity stratification. The study tracked the daily vertical distribution of *Dinophysis* spp. cells and their ciliate prey, *Mesodinium* cf. *rubrum*, and quantified the amount of lipophilic toxins present in seston and accumulated by various marine organisms in the food web. The abundance of the *D. acuminata* complex reached 43 × 10^4^ cells·L^−1^ at 1.0 m depth at the peak of the bloom. Maximum cell densities of cryptophyceans and *M.* cf. *rubrum* (>500 × 10^4^ and 18 × 10^4^ cell·L^−1^, respectively) were recorded on the first day of sampling, one week before the peak in abundance of the *D. acuminata* complex. The diarrheic toxin okadaic acid (OA) was the only toxin detected during the bloom, attaining unprecedented, high concentrations of up to 829 µg·L^−1^ in seston, and 143 ± 93 pg·cell^−1^ in individually picked cells of the *D. acuminata* complex. Suspension-feeders such as the mussel, *Perna perna*, and barnacle, *Megabalanus tintinnabulum*, accumulated maximum OA levels (up to 578.4 and 21.9 µg total OA·Kg^−1^, respectively) during early bloom stages, whereas predators and detritivores such as Caprellidae amphipods (154.6 µg·Kg^−1^), *Stramonita haemastoma* gastropods (111.6 µg·Kg^−1^), *Pilumnus spinosissimus* crabs (33.4 µg·Kg^−1^) and a commercially important species of shrimp, *Xiphopenaeus kroyeri* (7.2 µg·Kg^−1^), only incorporated OA from mid- to late bloom stages. Conjugated forms of OA were dominant (>70%) in most organisms, except in blenny fish, *Hypleurochilus fissicornis*, and polychaetes, *Pseudonereis palpata* (up to 59.3 and 164.6 µg total OA·Kg^−1^, respectively), which contained mostly free-OA throughout the bloom. Although algal toxins are only regulated in bivalves during toxic blooms in most countries, including Brazil, this study indicates that human seafood consumers might be exposed to moderate toxin levels from a variety of other vectors during intense toxic outbreaks.

## 1. Introduction

The frequency, duration and severity of *Dinophysis* blooms have increased worldwide over the past two decades [1], leading to numerous episodes of massive shellfish contamination by lipophilic toxins in Europe [2,3], Africa [4,5], Asia [6], North America [7], and South America [8,9,10]. Although scientific evidence indicates that the increase in harmful algal blooms may be correlated with meso- and large-scale physico-chemical processes, i.e., artificial eutrophication [11,12,13,14], and global climate change [15,16], possible causes for an apparent increase in *Dinophysis* blooms are less comprehended.

Neritic and oceanic *Dinophysis* spp. are frequently observed in offshore waters along the southern coast of Brazil [17]. In 2007, the first large-scale bloom of *Dinophysis acuminata* complex ever reported in this region caused intoxication of at least 170 human consumers of contaminated shellfish (mainly *Perna perna* mussels [18,19]) and led managers and regulators to issue a first-time ban for bivalve mollusk harvesting and commercialization. Recurrent small to medium-scale *Dinophysis* blooms in Brazil have been reported along the coasts of Paraná and Santa Catarina states since then [20,21]. In many cases, episodes of bivalve contamination have been reported based on Diarrheic Shellfish Poisoning (DSP) mouse bioassays [18,22]. Additionally, diarrheic toxins such as okadaic acid (OA) and their congeners dinophysistoxins (DTXs) have been detected by chemical analytical methods in plankton and marine fauna [20,22]. The current Brazilian national monitoring program for harmful algae and phycotoxins uses bivalves (especially brown mussels, *Perna perna*) as sentinel organisms for the presence of toxins in shellfish farming areas, and harvesting bans are issue anytime the regulatory toxin levels are surpassed, i.e., 160 µg·Kg^−1^ in the case of diarrheic toxins [23].

*Dinophysis* spp. are a recurrent threat to shellfish aquaculture areas worldwide (reviewed by Reguera et al., [24]), where bloom initiation depends not only on favorable abiotic conditions, but also on the availability of ciliate prey [25]. Mixotrophy via the sequestration and retention of plastids from the ciliate *Mesodinium* cf. *rubrum* is now considered a key process enabling the development of *Dinophysis* populations both in the laboratory and in the field (e.g., [26,27]). Recent studies have focused on describing the feeding mechanism of *Dinophysis* spp., and elucidating the possible ecological roles of diarrheic toxins and other bioactives produced by the dinoflagellate [27,28,29]. A number of nutritional and trophic aspects related to toxic *Dinophysis* spp. blooms, such as small-scale interactions with *M.* cf. *rubrum* and possibly with other prey items in the field, however, remain unclear.

During *Dinophysis* blooms, lipophilic toxins can be transferred via several trophic pathways [30]. Toxins can be accumulated not only by bivalves, but also by polychaetes and ascidians [31], fish [32,33], octopuses [20] and crabs [34,35]. *Dinophysis* toxins may also be related to the death of monk seals off the coast of the western Sahara [36], although the implications of toxin incorporation for marine organisms remain poorly known. An understanding of small-scale trophic relationships underlying the initiation and development of *Dinophysis* blooms, as well as the fate of diarrheic toxins in marine food webs, are essential for the evaluation of their associated risks. The main objectives of this study are to (a) determine the diel vertical distribution of *Dinophysis* spp. and their prey in a shallow inlet, and (b) quantify the levels of lipophilic toxins present in seston and in marine organisms representative of different trophic levels, during a massive bloom of the *Dinophysis acuminata* complex in southern Brazil.

## 2. Results

### 2.1. Plankton and Toxins in the Water Column

**Figure 1:**
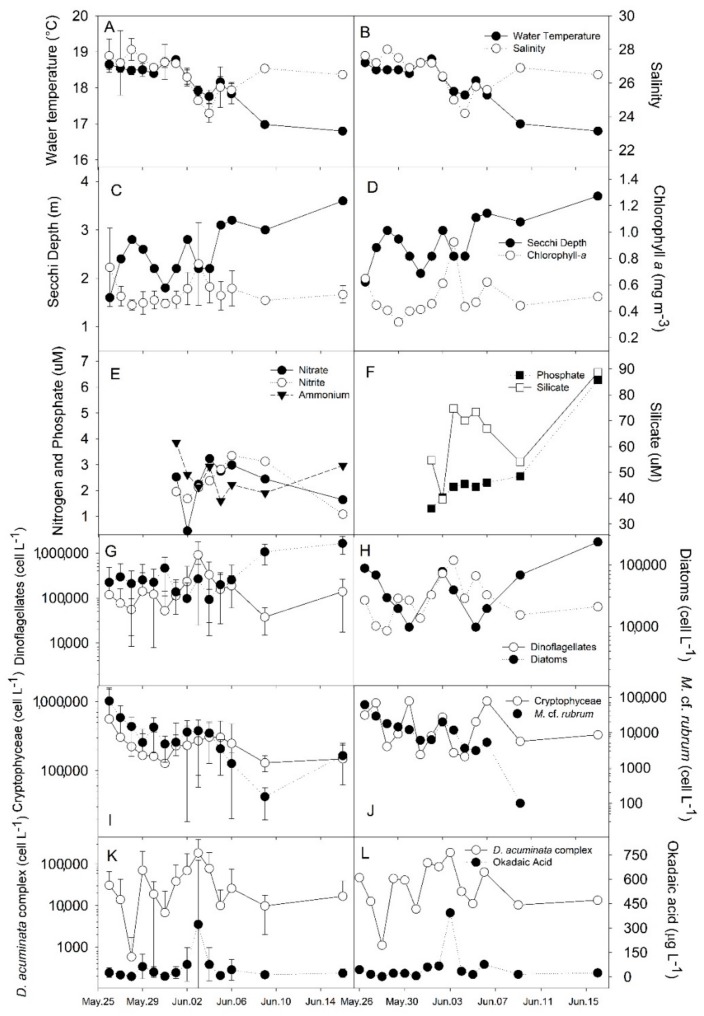
(**A**,**C**,**G**,**I**,**K**) Average values (± standard deviation; *n* = 6) of depth-discrete measurements, and (**B**,**D**,**E**,**F**,**H**,**J**,**L**) single depth-integrated measurements (taken with a hose extending from the surface to the bottom) for: (**A**,**B**) water temperature (°C) and salinity ; (**C**,**D**) Secchi depth (m) and chlorophyll-*a* concentration (mg·m^−3^); (**E**,**F**) concentration of dissolved inorganic nutrients (µM); (**G**,**H**) numerical abundance (on log-scale) of dinoflagellates and diatoms (cells·L^−1^); (**I**,**J**) abundance of cryptophyceans and *Mesodinium* cf. *rubrum* (cells·L^−1^); (**K**,**L**) abundance of the *Dinophysis acuminata* spp. complex (cells·L^−1^) and concentration of free okadaic acid (OA) in suspended particulate matter (µg·L^−1^).

Depth-averaged water temperature and salinity decreased gradually during the first half of the bloom period, from 26 May to 3 June, when the minimum salinities were recorded (mean ± standard deviation (SD) = 24.2 ± 0.55; *n* = 6). The water temperature continued to decrease thereafter, attaining a minimum of 16.8 ± 0.1 °C on the last sampling day, 16 June (Figure 1A,B). Secchi depth (Figure 1C,D) ranged from 1.8 to 2.8 m during the first half of the bloom and then gradually increased, attaining up to 3.6 m by the end of bloom. Chlorophyll-*a* concentrations were relatively low (0.47 ± 0.17 SD mg·m^−3^) throughout the study, and reached a maximum of 1.1 mg·m^−3^ on 3 June, coinciding with the maximum peak of *Dinophysis* abundance. Decreasing concentrations of mean DIN (±SD), especially those of nitrate (2.3 ± 0.9 µM) and ammonium (2.5 ± 0.7 µM) (Figure 1E), were associated with a concurrent decrease in salinity and temperature, and an increase in the abundance of *M.* cf. *rubrum*. Silicate and phosphate exhibited a marked increase during later stages of the bloom and attained the highest concentration range (39.6–88.7 µM and 1.3–6.3 µM, respectively) by the end of the study period (Figure 1F).

Diatom abundance remained at low to moderate levels (<9 × 10^4^ cells·L^−1^) during the first half of the bloom, rising up to 19 ± 6.2 × 10^4^ cells·L^−1^ by the end of the sampling period, when they finally dominated the micro-phytoplankton assemblage (Figure 1H). Dinoflagellates were detected at cell densities comparable to those of diatoms during the first half of the study, attaining a maximum of 90 ± 31.9 × 10^4^ cells·L^−1^ and becoming dominant over diatoms on 3 June (Figure 1H). *Dinophysis* species found during this bloom included the taxonomic complex composed by *D. acuminata* and *D. ovum* (referred to as *D. acuminata* complex hereafter), and *D. caudata*. This last species was frequently observed in plankton net samples during the first half of the bloom, although no cells were detected in most cell counts (LOD: 50 cells·L^−1^), except on 28 and 30 May (100 cells·L^−1^; not shown). The *Dinophysis acuminata* complex comprised the most abundant dinoflagellate cells and the main component of the total micro-phytoplankton assemblage throughout the study. Their depth-averaged cell density increased from 7.1 ± 13.4 × 10^4^ cells·L^−1^ on the first sampling day to 18.7 ± 20.1 × 10^4^ cells·L^−1^ (max. 43 × 10^4^ cells·L^−1^ at 1.0 m depth) on 3 June (Figure 1K), coinciding with a gradual decrease in the abundance of the ciliate *M.* cf. *rubrum* (Figure 1I). At the beginning of the bloom, average cell densities of cryptophyceans decreased at a rate comparable to that of *M.* cf. *rubrum*, and then increased slightly by the mid-bloom period, when *M.* cf. *rubrum* abundance reached minimum values (Figure 1I).

Water temperature (Kruskal-Wallis test statistic H = 69.4; *p* = 0.01), Secchi depth (H = 79.0; *p* = 0.01), salinity (H = 55.5; *p* = 0.01) and abundance of diatoms (H = 34.3; *p* = 0.01) all varied significantly during the study period. When depth layers were compared over time, however, there was no detectable difference in water temperature (H = 0.24; *p* = 0.99) or diatom abundance values (H = 6.6; *p* = 0.25) over time at any specific depth. The depth layer marking salinity stratification (H = 14.8; *p* = 0.01) ranged from 2 to 3 m over the course of the bloom. Although peaks in the abundance of targeted taxa were clearly identified in time and space/depth, there were no statistically significant differences in the cell density of dinoflagellates, cryptophyceans, *M.* cf. *rubrum* and the *D. acuminata* complex, or in the concentrations of chl-*a* and OA in SPM over time. Chlorophyll-*a* concentrations (H = 45.0; *p* < 0.01), and the abundance of dinoflagellates (H = 51.7; *p* < 0.01), cryptophyceans (H = 56.4; *p* < 0.01), *M.* cf. *rubrum* (H = 31.6; *p* < 0.01), and the *D. acuminata* complex (H = 53.4; *p* < 0.01) all differed significantly between 0–2 m and 3–5 m depth, with higher values found in the upper water layer. In general, depth-integrated samples (those taken with a hose extending from surface to bottom), yielded very similar values to those calculated as the average of depth-discrete measurements, except for the abundance of cryptophyceans (H = 20.3; *p* < 0.01), which was typically greater when estimated from integrated samples (Figure 1I,J).

**Figure 2:**
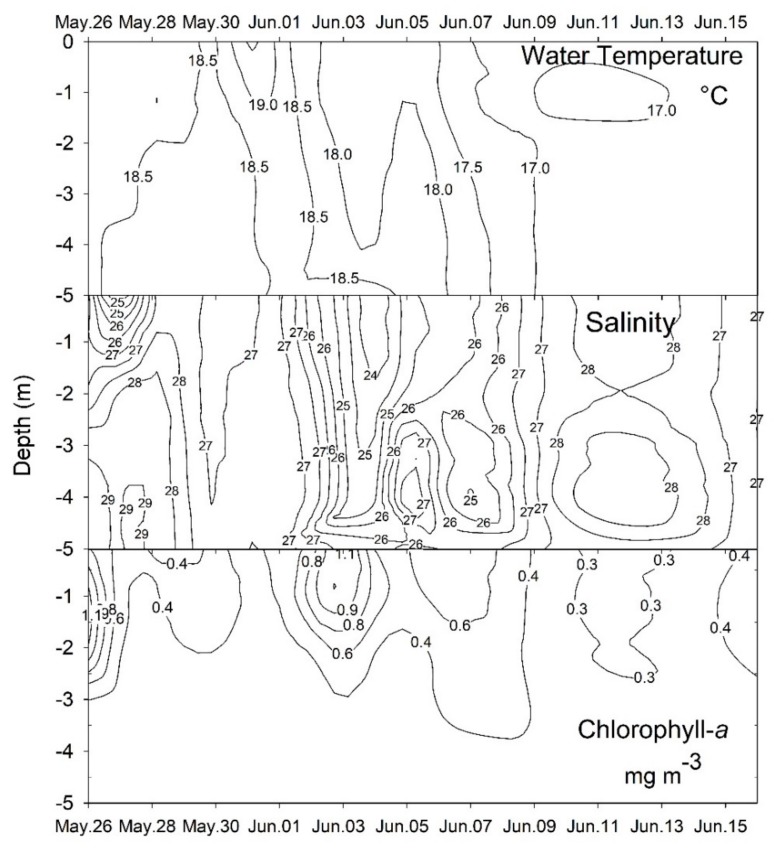
Interpolated depth-discrete measurements of water temperature (°C), salinity, and chlorophyll *a* concentration (mg·m^−3^), over the course of the study.

The onset of the bloom (on 26 May) was marked by pronounced surface stratification in salinity and water temperatures around 18 °C (Figure 2). After 2–3 days, salinity stratification was disrupted and the temperature increased by 1.0 °C, decreasing thereafter to a minimum of 17 °C by the 10th day (4 June), when the water column again became salinity-stratified (Figure 2). Water temperature ranged from 17 to 19.5 °C and salinity from 23 to 29 over time. The relatively high Secchi-depth values (>2.0 m) obtained during the study indicate that the euphotic zone always reached the bottom in the sampling area.

**Figure 3:**
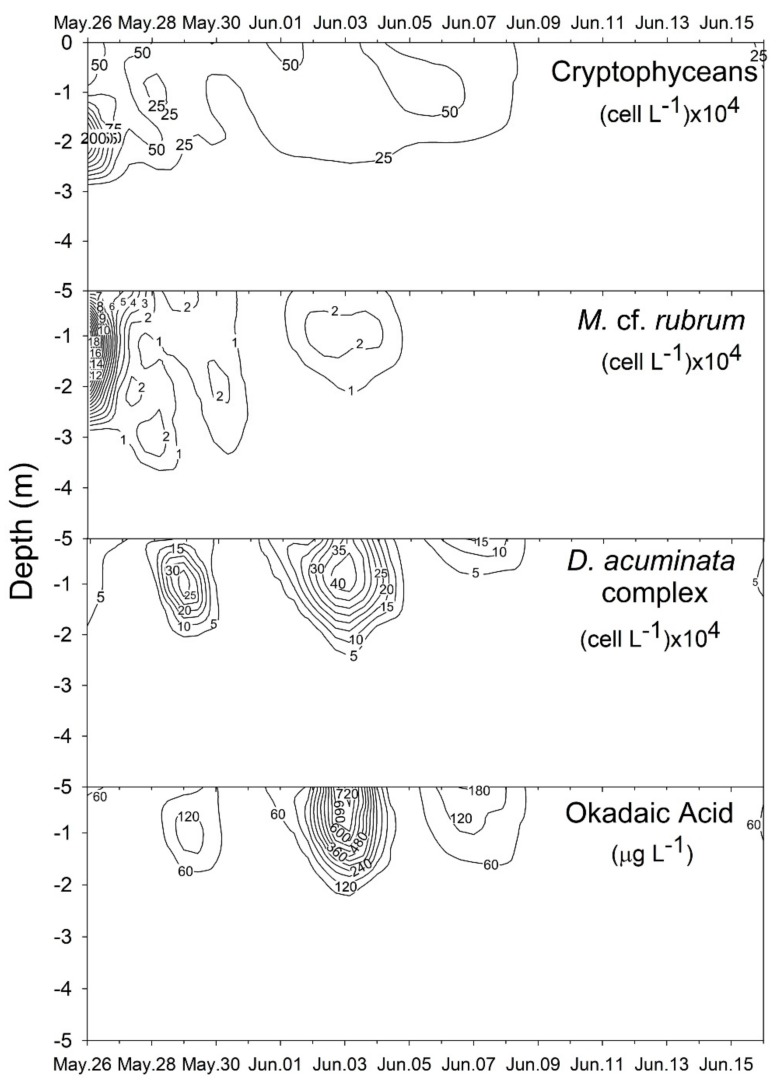
Depth profile of the cell abundance of the main plankton taxonomic groups (cell·L^−1^ × 10^4^) (top three panels) and the concentration of okadaic acid (µg·L^−1^) in suspended particulate matter (bottom panel) during the study period.

Chlorophyll-*a* concentrations were higher during periods of salinity stratification. Values ≥1.0 mg·m^−3^ were attained on two occasions, on the 1st and the 9th day of sampling (26 May and 3 June), with a third peak (>0.6 mg·m^−3^) a few days later (Figure 1D and Figure 2). In all cases, higher concentrations were restricted (H = 0.45; *p* < 0.01) to the surface layer (0–2 m). Likewise, the abundance of the main taxa investigated–cryptophyceans, *M.* cf. *rubrum* and *Dinophysis*–also varied vertically, exhibiting higher values in the upper water layer. Maximum values for cryptophyceans and *M.* cf. *rubrum* (>500 × 10^4^ and 18 × 10^4^ cell·L^−1^, respectively) were measured on the first day of sampling. During subsequent days, their abundance decreased concomitantly with a rapid increase of the *D. acuminata* complex cell density, reaching >20 × 10^4^ cells·L^−1^ on 29 May and >40 × 10^4^ cells·L^−1^ on 3 June, one week following the initial peak in *M.* cf. *rubrum* abundance (Figure 3), and coincident with the second episode of salinity stratification. Higher cell abundances of the *D. acuminata* complex were restricted to the surface layer (>2 m), where concentrations of free-OA >600 µg·L^−1^ were simultaneously detected in the SPM (>0.45-µm particles). During the period of maximum dinoflagellate cell density, the OA cellular quota, as measured from individually-picked cells of the *D. acuminata* complex ranged from 48 ± 31 SD pg·cells^−1^ (*n* = 137–208 cells per sample) on 2 June to 143 ± 93 pg·cell^−1^ (*n* = 141–220) on 3 June.

### 2.2. Diarrheic Toxins in Marine Fauna

**Figure 4:**
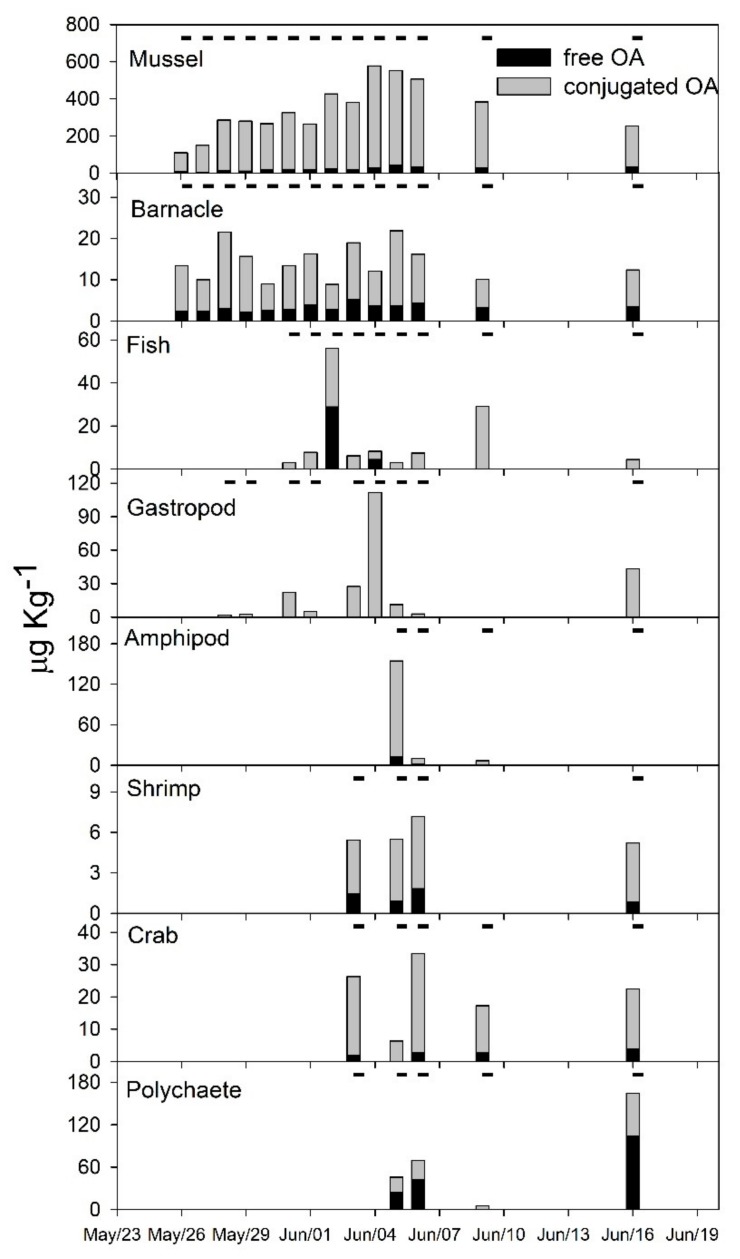
Concentration of okadaic acid (OA, µg·kg^−1^), in its free (black bars) and conjugated (gray bars) forms, accumulated in different marine organisms during the bloom of the *Dinophysis acuminata* spp. complex. Dashes above the composite bars denote the sampling dates when each marine organism was available.

All selected marine faunal components accumulated detectable OA levels during the study. Toxin levels were directly associated with the presence of *D. acuminata* complex cells in the water column; maximum values depended on the species and trophic position of the organisms. Suspension-feeding mussels and barnacles were the first to accumulate detectable levels of OA in their tissues and the only ones to contain detectable toxin levels during the entire sampling period. Mussels accumulated the highest OA concentrations among all organisms analyzed, with toxin levels gradually increasing following an increase in cell density of the *D. acuminata* complex. They attained a maximum of 549.6 µg total OA·Kg^−1^ (wet tissue weight) on 4 June (Figure 4), only one day after the peak abundance of the dinoflagellate. Polychaete worms (max. = 164.5 µg total OA·Kg^−1^), amphipods (max. = 153.7 µg total OA·Kg^−1^) and gastropods (max. = 111.6 µg total OA·Kg^−1^) also retained relatively high toxin amounts, but not before the mid-bloom stage. Amphipods and to some extent fish (max. = 56.2 µg total OA·Kg^−1^), remained contaminated for shorter periods, i.e., only when OA concentrations in the SPM were maximal (early to mid-bloom period). In contrast, gastropods, polychaetes, crabs (max. = 33.3 µg total OA·Kg^−1^) and shrimp (max. = 7.2 µg total OA·Kg^−1^) accumulated detectable toxin levels from the mid- to late bloom stage, after OA peaked in suspension (Figure 4).

Fish and polychaetes accumulated the greatest proportions of OA in its free form. The proportion of free-OA was slightly higher in fish (54 ± 7%) at the peak of the bloom, but still did not match that of polychaetes (64 ± 5%). The latter seemed to possess the least efficient detoxification mechanism, given the high proportions of free-OA and the constantly increasing total OA levels measured (Figure 4). Conversely, the conjugated forms of OA were dominant in all other organisms, including barnacles (76 ± 7% SD), shrimp (79 ± 5%), amphipods (87 ± 5%), crabs (90 ± 7%), mussels (94 ± 2%) and gastropods, which never accumulated detectable levels of free-OA (i.e., exhibited 100% of OA as conjugated forms).

### 2.3. Correlations

**Figure 5:**
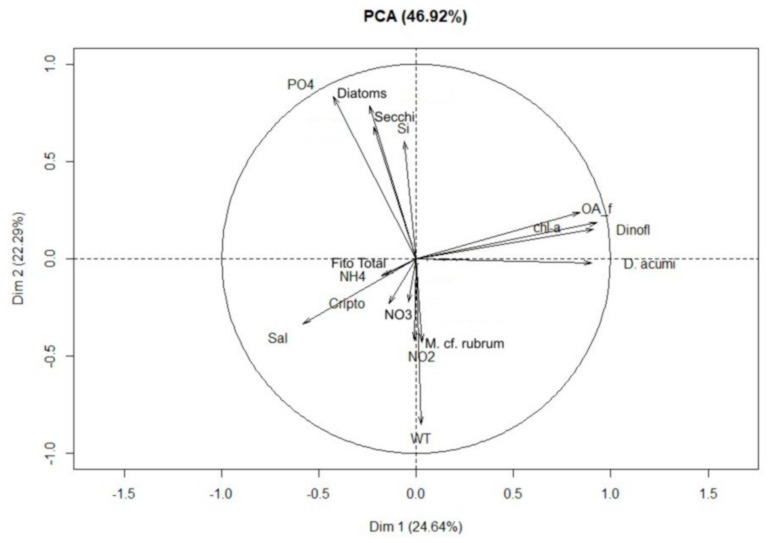
Principal Component Analysis (PCA) of discrete-depth measurements of the following variables: water temperature (WT), Salinity (Sal), Transparency (Secchi depth), Chlorophyll-*a* (chl-*a*), Diatoms (Diatoms), Dinoflagellates (Dinofl), Cryptophyceans (Cripto), total micro-phytoplankton (Fito Total), *Mesodinium* cf. *rubrum* (*M.* cf. *rubrum*), *D. acuminata* complex (*D. acumi*), free okadaic acid (AO_f), Phosphate (PO4), Nitrate (NO3), Nitrite (NO2), Ammonium (NH4), and Silicate (Si) concentrations.

As assessed by principal component analysis, salinity was strongly and inversely correlated with both the abundance of the *D. acuminata* complex and the concentration of OA in suspension (Figure 5). This grouping was also inversely, but less obviously associated with ammonium concentration and the abundance of cryptophyceans, and even less obviously with Secchi depth, phosphate concentration and the abundance of diatoms (Figure 5). These last three variables, as well as the concentration of silicate, were inversely correlated to *M.* cf. *rubrum* abundance, which, in turn, was strongly and directly associated with the abundance of cryptophyceans and the concentrations of nitrite and nitrate.

## 3. Discussion

### 3.1. Bloom Development and Trophic Relationships

In late May 2016, an episode of marked salinity stratification was associated with the onset of what can be considered the most intense bloom of the *Dinophysis acuminata* complex ever recorded along the Brazilian coast. The bloom only lasted for a few weeks along the coast of Santa Catarina State. It was then transported northward to Paraná State, where it reached even higher cell densities, causing massive contamination of marine fauna and intoxication of human seafood consumers [37]. Although results of the present study indicated that bivalves were also contaminated with unsafe OA levels in Santa Catarina, actions taken in the context of the local HAB monitoring and management program prevented cases of intoxication in this region. More importantly, however, the present study also documents the accumulation of diarrheic toxins in several other marine organisms associated with farmed mussels, some of them for the first time, indicating that multiple toxin vectors and transfer routes should be considered during massive *Dinophysis* blooms.

Blooms of *Dinophysis* spp. are usually associated with marked thermohaline stratification of the water column [10,38,39,40]. The ciliate prey of *Dinophysis* spp., frequently reported as *Mesodinium rubrum*, usually benefits from vertical water stratification as well [41,42], although blooms of the ciliate may also occur along horizontal thermohaline gradients in shallow estuaries [43].

About one week preceding the maximum *Dinophysis* cell density recorded in Armação do Itapocoroy inlet during this study, high abundances of *M.* cf. *rubrum* and their cryptophycean prey (10^5^ to 10^6^ cells·L^−1^, respectively) were observed in the upper water layer associated with lower salinities and strong stratification at 2 m depth. On the following 4–5 days, the abundance of cryptophyceans decreased rapidly, followed by a more gradual decrease in *M.* cf. *rubrum* cell density, as the abundance of cells belonging to the *D. acuminata* complex began to increase in the same surface layer. One week later, a second cryptophycean-ciliate-*Dinophysis* succession cycle occurred once water became stratified again. Although daily variations in the abundance of these three taxa may be partially linked to local advection, what was not the subject of this study; the succession pattern reported herein confirms that the trophic relationships documented in prior laboratory observations [27,28,44,45,46] may also occur on a similar temporal scale under natural field conditions and sustain massive blooms of the toxic dinoflagellate. On other occasions (i.e., under lower availability of *M.* cf. *rubrum* cells), alternative prey items may provide *Dinophysis* spp. with an additional source of nutrients, as suggested for *D. caudata* preying upon the benthic ciliate *Mesodinium coatsi* [47].

The development of *Dinophysis* blooms in other geographical areas may also be linked to the intrusion of less saline water masses and/or to disturbances in physico-chemical water column structure, although the underlying processes might be different and sometimes occur on a wider spatio-temporal scale. Blooms may thus be either associated to upwelling, as verified in Sweden [48], Galicia (Spain) and Portugal [25], or to river plumes, as found in Tunisia [40] and Scotland [3]. They may also be associated with seasonal changes in wind patterns and the precipitation regime such as those recorded in Ireland [49], Greece [50] and Argentina [9,51]. On the eastern coast of South America, the water mass associated with the La Plata River plume promotes important large-scale changes in the physico-chemical characteristics of the water column along the coasts of NE Argentina, Uruguay and southern Brazil during fall and winter [52,53], when massive *Dinophysis* blooms are usually observed in this region [54,55]. This suggests that the La Plata water plume (PWP) may be one of the main factors controlling the development of large-scale *Dinophysis* blooms in southwestern Atlantic coastal waters.

Chlorophyll-*a* concentrations did not vary substantially over time in the present study, and did not attain values exceeding the historical average for the region [56,57]. This could be explained by the uncommon prevailing phytoplankton succession, whereby one dominant taxon preys upon and acquires the plastids (and the pigments) from its precursor, rather than synthesizing its own pigment quota during a gradual competitive exclusion process. Whereas phosphate concentrations remained relatively high and even increased during the final bloom stage, those of dissolved nitrogen compounds, notably ammonium and nitrate, decreased over the course of the bloom, especially during the period of maximum cell abundance of the *D. acuminata* complex.

Both water sampling strategies used in this study (single integrated samples and multiple depth-discrete sampling) allowed adequate tracking of bloom development, yielding similar abundance values for both the toxic dinoflagellate and its prey, *M.* cf. *rubrum*. Therefore, as demonstrated in other areas such as Spain [58], depth-integrated sampling, undertaken with a hose extending from the surface to the bottom, provided a rapid and reliable early-warning tool in HAB monitoring and risk assessment in shallow waters affected by *Dinophysis* blooms along the southern coast of Brazil. However, special attention is required when applying the technique to ecological studies, as the abundance of cryptophyceans and perhaps other small-celled algal groups can be underestimated. Likewise, vertical migration and cell aggregation processes can be missed as a result of the “diluting” effect introduced by this sampling strategy. More importantly, adoption of *Dinophysis* cell abundance as early warning for DSP should be used conservatively, i.e., the threshold value should be kept cautiously low when integrated samples are used in HAB monitoring programs. One of the main ecological features of *Dinophysis* cells is their ability to aggregate in thin water layers, as reported in this and other studies [59], such that toxin food web transfer may be heterogeneous throughout the water column.

### 3.2. Fate of Diarrheic Toxins during the Bloom

Cells of the *D. acuminata* complex contained exclusively OA during the bloom described in this study, contrasting with a more complex toxin profile reported during previous blooms in Argentina [10,60] and Chile [61], where *D. acuminata* and *D. tripos*, the species involved in the blooms, produced pectenotoxin-2 (PTX-2) and DTX-1 in addition to OA. It is noteworthy that DTX-1 has been reported in different southern Brazilian estuaries when lower *Dinophysis* spp. cell abundances (<2 × 10^4^ cell·L^−1^) occur, but rarely when only cells of the *D. acuminata* complex are detected, in which case OA usually becomes the single diarrheic toxin present [32]. Similarly, in late summer 2015, one year before the event reported in this study, an extremely dense bloom of the *Dinophysis acuminata* complex affected the coast of Uruguay and only OA was detected by LC-MS/MS [32]. This further suggests that there may be interconnectivity between Uruguayan and southern Brazilian populations of the *D. acuminata* complex, perhaps driven by the northward transport of PWP from late summer to winter. This possibility remains to be addressed in future studies.

All marine organisms collected in the upper water layer (0–1 m) at the sampling site were consistently contaminated with varying amounts of OA. This is the first record of diarrheic toxin accumulation in amphipods (Caprellidae), shrimp (*X. kroyeri*), Nereidae polychaetes and blenny fish (Blenniedae). The only previous records of OA content in fish included carnivorous flounders, *Platichthys flesus* [33], and filter-feeding anchovies, *Cetengraulis edentulus* [32]. Results of the present study demonstrated that the combtooth blenny, *Hypleurochilus fissicornis*, can accumulate moderate OA levels in their viscera during *Dinophysis* blooms, but are able to rapidly eliminate the toxin. Therefore, this fish species may act as a temporary vector of diarrheic toxins for other species, including commercially important species of Serranidae and Lutjanidae, which prey upon small fish like blennies [62]. *Hypleurochilus fissicornis* is widely distributed in the southwest Atlantic; adults feed primarily on isopods and amphipods [63], that were likely an important–although probably not the sole–toxin source for the fish during the bloom, as the peak in OA levels occurred later for amphipods than for *H. fissicornis* in the present study. Amphipods accumulated relatively high OA levels (up to ~150 µg OA·Kg^−1^) at the peak of the bloom. Although most amphipods are detritivores/scavengers, caprellids such as the ones sampled in our study are omnivorous and may feed not only on detritus, but also on microalgae, protozoans, smaller amphipods and crustacean larvae [64]. Caprellids are frequent and abundant organisms associated with suspended mussel farms, living on substrates such as mussel sleeves and ropes, and may thus be important toxin vectors to several organisms that search for shelter and food within the mussel longlines in aquaculture areas.

Mussels accumulated the greatest OA levels during the bloom, exceeding by 4-fold the 160-µg·Kg^−1^ Brazilian regulatory seafood safety level [65]. *Perna perna* mussels are the sentinel species in HAB monitoring programs in Brazil and, like other mussel species, are able to rapidly incorporate high levels of several marine biotoxins and contaminants [66,67,68,69,70]. Indeed, along with barnacles, mussels were the only organisms exhibiting detectable OA levels during the entire sampling period. They consistently and promptly reflected the abundance of the *D. acuminata* complex (i.e., they attained maximum OA levels only one day after the peak in cell abundance). Considering the high *Dinophysis* cell abundance reported during this bloom, however, OA levels in *P. perna* were not as high as expected, what may be related to its fast toxin elimination rates as reported in previous laboratory experiments [21]. Besides mussels, non-edible polychaetes (*P. palpata*) and amphipods were the only organisms to accumulate total OA levels approaching or surpassing this regulatory level in the present study. Toxin contents in barnacles were 8 to 48 × lower–and less clearly related to *Dinophysis* cell density–than those of mussels. Barnacles colonize hard substrates, rocks, bivalve shells and mooring structures, living in clusters of around a dozen individuals that actively capture food particles from the surrounding water [71]. In the present study, barnacles were collected from the shells of the same mussels sampled for OA analysis, so that the differential toxin accumulation reported here for these two suspension-feeding taxa can only be attributed to distinct feeding mechanisms and toxin uptake/elimination capacity. The consistently greater proportions of conjugated OA in mussels (>90%) reflect their efficient mechanisms of toxin metabolism and elimination. Likewise, Caprellidae amphipods, small crabs (*P. spinosissimus*), shrimp (*X. kroyeri*) may have ingested toxins from the grazers or their organic matter produced [72,73], and carnivorous gastropods (*S. haemastoma*) also accumulated very limited to undetectable free-OA levels. This finding at least partly suggests that these organisms ingested already metabolized (i.e., conjugated) toxin, either incorporated into mussel and barnacle tissues (gastropods, shrimp and crabs) or from detrital origin (in the case of amphipods and crabs). High toxin levels were found in seston during this study and in particles >60 µm (L. Mafra, unpublished data), suggesting that not only *Dinophysis* cells but also toxin-containing organic particles and zooplankton organisms may contribute to the transfer of diarrheic toxins along the foodweb. Therefore, although zooplankton (e.g., copepods) may exert significant grazing impact and contribute considerably to control population growth of *Dinophysis* spp. [74], contaminated individuals will act as vectors of DSP-toxins to higher trophic levels.

Transfer of lipophilic toxins in the marine food web is still poorly understood. Although they represent only a small fraction of the sinking organic material, zooplankters such as the copepod *Temora longicornis*, might contribute in maintaining toxin availability for other organisms via production of toxic faecal pellets following ingestion of *Dinophysis* cells [73]. Inter- and intraspecific differences in the capacity of uptake and elimination of phycotoxins, as reported for suspension-feeding grazers such as oysters, clams and mussels [21,75,76,77], may ultimately determine the bioavailability of these compounds for other organisms during and after a bloom. In this study, polychaetes, which exhibited the highest proportions of free-OA and whose total OA levels continued to increase through the end of the sampling period, proved to be slow in eliminating OA. They may thus be an important toxin vector during late bloom stages, by prolonging toxin availability in the trophic web even after bloom termination.

To date, potential vectors of diarrheic toxins to human consumers have been restricted to several bivalve species (reviewed by FAO/WHO [78]), a couple of fish species [32,33], octopuses [20] and crabs [34,35]. The present study indicates that seabob shrimp (*X. kroyeri*) can represent a novel vector for toxin transfer to humans during massive dinoflagellate blooms. Although these shrimp accumulated the lowest OA levels (≤7 µg·Kg^−1^) of all investigated faunal species and thus cannot be classified as a risk for acute food intoxication among seafood consumers, frequent consumers of this valuable fishery resource may be chronically exposed to low toxin levels during prolonged blooms. Seabob shrimp catches may reach 170 tons per year only in the Armação do Itapocoroy area [79], our study site, and the seabob shrimp fishing season coincides with the usual period of *Dinophysis* blooms in southern Brazil (winter to spring; [80]). Additionally, shrimp and, to some extent crabs and blenny fish, are highly motile. Their frequent vertical migration throughout the water column or on mussel ropes and sleeves may thus help to accelerate toxin transfer from pelagic to benthic compartments, and to spread diarrheic toxins over a more complex trophic web.

## 4. Materials and Methods

### 4.1. Study Area

**Figure 6:**
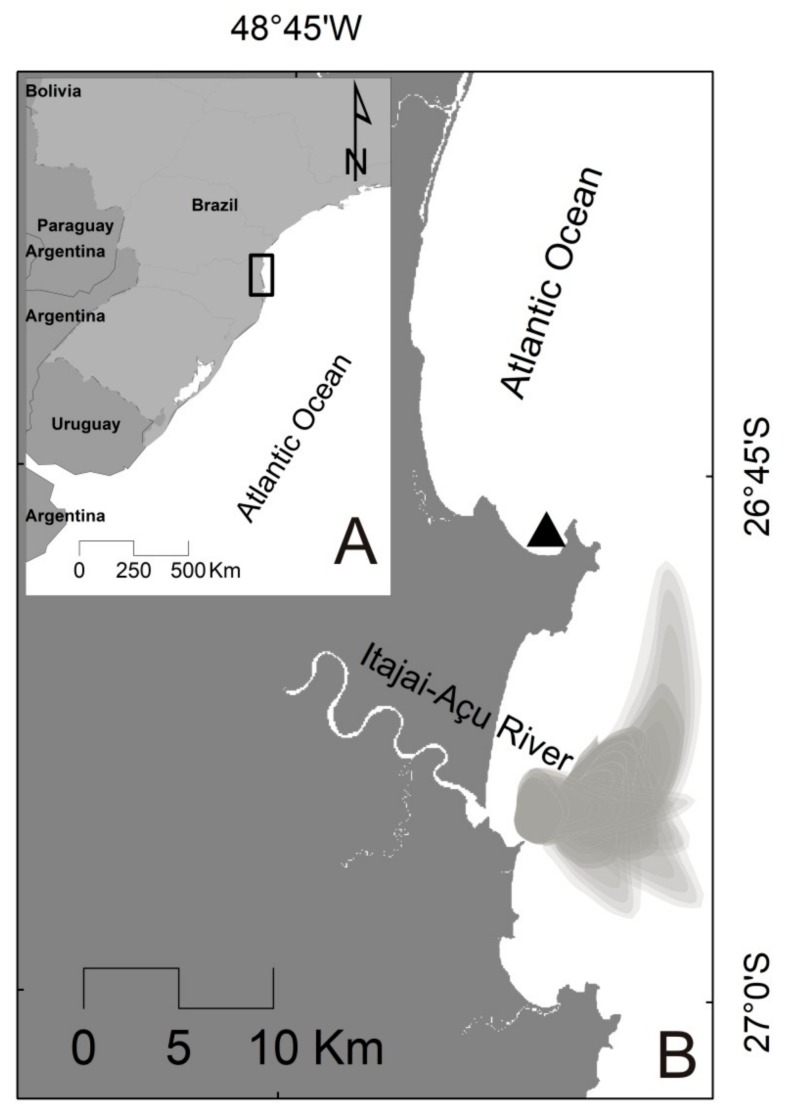
Map showing the location of the study area, Armação do Itapocoroy inlet (triangle in (**B**)), on the southern coast of Brazil (rectangle in (**A**)). A schematic representation of the prevailing direction and influence area of the Itajaí-Açu River plume is also presented, based on Trochimczuk-Fo and Schettini (2003) [83].

Armação do Itapocoroy is a shallow [mean depth = 8 m; maximum (max.) = 15 m] inlet in Santa Catarina State, southern Brazil (26°47′ S, 48°37′ W). Surrounded by hills (up to 250 m high), its geographic SE-NE orientation provides natural shelter from prevailing waves and winds, especially the stronger ones coming from the south. Due to these favorable attributes, Armação do Itapocoroy (Figure 6) harbors the major marine aquaculture (~360 hectares) operations in the country, mainly used for the cultivation of mussels, but also of oysters and scallops. The location experiences semi-diurnal, micro-tidal cycles and is affected by the Itajaí-Açu River plume, that maintains high levels of local primary production and brings high loads of suspended particulate matter (SPM) mainly during rainy periods such as the austral summer (December–March) [81,82,83].

### 4.2. Sampling Design

An intensive sampling effort was conducted between late May and mid-June 2016, when the Santa Catarina coastal zone was affected by a dense *Dinophysis* bloom. Seawater sampling was carried out from a floating platform, anchored at a depth of 4.5 ± 0.5 m and deployed 200 m from the low tide level. Samples (~1.5 L) were taken every meter along a vertical profile, from the surface to the bottom, using an EN-470 manual diaphragm pump (Emifran^®^, São Paulo, SP, Brazil) equipped with anti-reflux valves and coupled to a 20 mm-diameter hose. Daily samples were taken over 12 days, followed by two sampling operations after 3- and 10-day intervals of the 12th sampling day. Additionally, single depth-integrated water samples (2.0 L) were taken with a 5 m long hose, to compare the efficiency of both sampling strategies for monitoring of extreme bloom events. Plankton net (20-µm mesh size) samples were also collected, and water temperature and salinity were measured along a vertical profile, at 0.5 m intervals from surface to bottom, using a multiparameter YSI Professional Plus probe. Secchi depth was used to estimate water column light penetration or transparency.

In parallel, bivalve mollusks (*Perna perna*; *n* > *5*), gastropods (*Stramonita haemastoma*; *n* > *2*), barnacles (*Megabalanus tintinnabulum*; *n* > *10*), amphipods (Caprellidae; ~10 g of wet weight), crabs (*Pilumnus spinosissimus*; *n* > *3*), shrimp (*Xiphopenaeus kroyeri*; *n* > *5*), polychaetes (*Pseudonereis palpate*; *n* > *3*) and fish (*Hypleurochilus fissicornis*–Blenniidae; *n* > 2) were manually collected from raft mooring cables at 0–1 m depth, for quantification of the toxin levels incorporated in their tissues. The species collected and sampling frequency depended on their availability in the environment during the study period. Whenever possible, at least two individuals of each species were collected, packed in 50 mL plastic tubes and immediately immersed in an ice bath until return to the laboratory. Before freezing, soft tissues of mussels, barnacles and gastropods were removed from their shells. Fish muscles (flesh) and viscera were dissected and individually stored at −18 °C. The remaining organisms were frozen and analyzed whole.

### 4.3. Processing of Samples

*Water samples*: Aliquots (300 mL) of both depth-discrete and integrated samples were fixed with 1% Lugol’s iodine solution, and used for quantitative phytoplankton analysis. Plankton net samples, fixed with a 4% formalin solution (final concentration), were used for analysis of cell morphology and phytoplankton identification. In the laboratory, additional 300 mL aliquots of each sample were gently vacuum-filtered in duplicate and immediately frozen. One fiberglass filter (Marcherey-Nagel^®^, Düren, Germany, model 85/70BF; 47 mm diameter and 0.45 µm nominal retention capacity) was allocated for the analysis of photosynthetic pigments, and the second one to determine the amount of lipophilic toxins contained in SPM. In an ice bath, each filter sample was soaked in HPLC grade methanol (99.5%) and exposed to an ultra-sonic probe (Cole Parmer, Vernon Hills, IL, USA, CPX130) for 30 s. The extract was then passed through a 13 mm × 0.22 µm PVDF syringe filter (Analitica^®^, São Paulo, SP, Brazil) to remove any cell debris, and the filtrate collected into plastic microtubes (1.5 mL), which were maintained frozen. Aliquots (400 mL) of the filtrate from depth-integrated samples collected from 1 May on were stored frozen in plastic bottles for future spectrophotometric determination of dissolved inorganic nutrient (DIN) concentrations.

*Samples of marine fauna*: The protocol for toxin extraction was adapted from the official analytical method harmonized by the European Union [84]. Methanol (99.5%; HPLC grade) was added in the ratio of 1:9 (*v*:*v*) to 1.0 ± 0.5 g of homogenate from selected tissues or whole body, exposed to an ultra-sonic probe (Cole Parmer, CPX130) until complete tissue disruption, and centrifuged at 2000× *g* for 10 min. The supernatant was filtered through a 0.22-µm syringe filter directly into a 2.0 mL glass vial, and kept frozen for the analysis of diarrheic toxins in their free form. Subsequently, 1 mL aliquots of the extract were subjected to alkaline hydrolysis by the addition of 2.5 M sodium hydroxide in a 76 °C thermal bath for 40 min, followed by the addition of 2.5 M hydrochloric acid to neutralize the solution and convert the conjugated (metabolized) toxins into their free toxin forms. The amount of conjugated toxins was obtained by subtracting the concentration of free toxins initially measured in the non-hydrolyzed extract from the total concentration of toxins obtained in the hydrolyzed extract.

### 4.4. Phytoplankton Identification and Enumeration

Counting of *Dinophysis* spp. and *M.* cf. *rubrum* cells was performed using a 20 mL aliquot of the Lugol-fixed sample, after settling the particles for 24 h in a Utermöhl chamber [85]. Cell counting was then performed by scanning the whole chamber under an inverted optical microscope at 200× magnification (limit of detection, LOD: 50 cell·L^−1^). Other phytoplankton groups (total cryptophytes, diatoms, and dinoflagellates) were quantified in volumes ranging from 10 to 20 mL (depending on sample turbidity) by counting all cells contained in 5–10 random microscope fields of view (LOD: 2100–8400 cell·L^−1^) after 24-h settlement. Additionally, a minimum of 100 cells of *Dinophysis* spp. were picked with a micropipette from *in natura* plankton net samples at the bloom apex (2 and 3 June; *n* = 5 samples each day), and placed into 1.5 mL plastic microtubes containing methanol 99.5% (HPLC grade) for determination of the toxin cellular quota.

### 4.5. Spectrophotometric Analysis of Dissolved Inorganic Nutrients

Aliquots (25 mL) of the filtrate samples were used to determine the concentrations of phosphate (P-PO_4_^3−^), nitrite (N-NO_2_^−^), nitrate (N-NO_3_^−^), ammonium (N-NH_4_^+^), and silicate (SiO_2_^−^) using colorimetric methods [86]. The concentrations were determined from a linear regression obtained from successive dilutions of the respective analytical standards (coefficient of determination, *r*^2^ > 0.90).

### 4.6. Analysis of Photosynthetic Pigments by LC-DAD

The methanolic extracts were injected (100 μL) into a Chromaster liquid chromatography (LC) system (Hitachi^®^, Tokyo, Japan), composed of a quaternary gradient pump, an automatic thermostat injector, a column oven (set at 40 °C) and a photodiode detector (DAD). Samples were eluted in a mixture of (A) methanol:acetone:pyridine (50:25:25) and (B) acetonitrile:acetone (80:20), at 1.0 mL·min^−1^. The proportion of B increased from 0 to 40% in 18 min, and then to 100% within the following 4 min of analysis, remaining at 100% for an extra 16 min before returning to the initial conditions (0% B) for an additional 2 min period (40 min in total). Pigment identification was performed by evaluating retention times after the chromatographic separation in a Waters Symmetry^®^ C8 column (150 × 4.6 mm, 3.5 μm particles), as well as the absorbance spectrum (350–750 nm scan), following methods of Zapata et al. [87]. The chlorophyll-*a* (chl-*a*) concentration was calculated using a linear regression obtained from successive dilutions of the analytical standard (Sigma-Aldrich, Saint Louis, MO, USA) (0.78, 1.56, 3.12, 6.25, 12.50, and 25.00 ng·mL^−1^) with *r*^2^ > 0.98.

### 4.7. Analysis of Diarrheic Toxins by LC-MS/MS

Toxins were measured using a 1260 LC system (Agilent Technologies^®^, Santa Clara, CA, USA) coupled to a triple quadrupole mass spectrometer, MS (AB Sciex^®^, Framingham, MA, USA, qTRAP 3200) equipped with a turbo ion spray ionization source, following the EURLMB protocol [84]. Briefly, 5 to 15 µL of each sample were eluted by the mobile phase, consisting of a mixture of (A) 100% ultra-pure water and (B) 95% acetonitrile, both with the addition of ammonium formate (2 mM) and formic acid (50 mM). At a 0.3 mL·min^−1^ flow rate, the initial proportion of 80:20% (A:B) increased to 100% B during the first 8 min of analysis, thus remaining for 3.5 min, and returning to the initial condition by the end of the analysis (13 min). Compounds were separated on a C18 column (Agilent Poroshell^®^, Santa Clara, CA, USA, 50 × 2.1 mm, 2.7 μm particles), maintained at 20 °C. Identification of individual toxins was achieved from their retention time and the mass spectra of the transition ions present in the samples in relation to the same parameters obtained for the analytical standards. High-purity nitrogen, heated up to 500 °C, was used as the nebulizing gas. The electron spray (ESI) ion source operated in negative mode, and toxins were scanned for transition ions (Q1 → Q3) of characteristic mass/charge (*m*/*z*) ratios. Optimized MS parameters were selected for each toxin of interest (Table 1).

Toxin quantification was carried out using an external standard from a calibration curve generated with certified reference material (IMB-NRC, Canada) dissolved in methanol for OA, and in mussel tissue matrix (CRM-DSP-Mus-b) for DTX-1. Quantification of OA was based on the equation obtained by fitting a linear regression (*r*^2^ > 0.95) to the following concentrations: 3.49, 13.96, 55.86, and 223.44 ng·mL^−1^.

### 4.8. Data Analysis

Graphs were constructed in SigmaPlot^®^ v11.0 (Systat Software Inc., London, UK), using the statistical package for preliminary analysis. Data were statistically analyzed with R studio software [88], using the Kruskal-Wallis non-parametric test (H) followed by the Dunn test (with the software package dunn.test; [89]) for comparative analyses within and among water column depths, between discrete and integrated samples, and for analysis of temporal variation. Principal components analysis (PCA) (with the FactoMiner package; [90]) allowed quantification of the degree of association among water temperature, Secchi depth, salinity, numerical abundance of main taxonomic groups, concentration of dissolved inorganic nutrients and seston toxin content.

## Figures and Tables

**Table 1:** Conditions of the tandem mass spectrometry system (MS/MS). Q1: quadrupole 1, Q3: quadrupole 3, DP: declustering potential, EP: entrance potential, CEP: collision cell entrance potential, CE: collision energy and CXP: collision cell exit potential.

Toxins	Q1 (*m*/*z*)	Q3 (*m*/*z*)	DP (v)	EP (v)	CEP (v)	CE (v)	CXP (v)
OA	803.5	255.0	−129	−10	−40.1	−82	−2
OA	803.5	113.0	−129	−10	−41.5	−64	−2
DTX-2	803.5	255.0	−129	−10	−40.6	−64	−2
DTX-2	803.5	113.0	−129	−10	−41.5	−84	−2
DTX-1	817.5	255.0	−129	−10	−41.5	−62	−2
DTX-1	817.5	113.0	−120	−10	−51.7	−82	−2
DTX-3	1041.6	255.0	−129	−10	−47.9	−76	−2
